# BODY IMAGE IN CHILDHOOD: AN INTEGRATIVE LITERATURE REVIEW

**DOI:** 10.1590/1984-0462/;2017;35;3;00002

**Published:** 2017-07-20

**Authors:** Clara Mockdece Neves, Flávia Marcelle Cipriani, Juliana Fernandes Filgueiras Meireles, Fabiane Frota da Rocha Morgado, Maria Elisa Caputo Ferreira

**Affiliations:** a Universidade Federal de Juiz de Fora (UFJF), Juiz de Fora, MG, Brasil.; b Universidade Federal Rural do Rio de Janeiro (UFRRJ), Rio de Janeiro, RJ, Brasil.

**Keywords:** Body image, Child, Feeding and eating disorders

## Abstract

**Objective:**

To analyse the scientific literature regarding the evaluation of body image in children through an integrative literature review.

**Data source:**

An intersection of the keywords “body image” AND “child” was conducted in Scopus, Medline and Virtual Health Library (BVS - *Biblioteca Virtual de Saúde*) databases. The electronic search was based on studies published from January 2013 to January 2016, in order to verify the most current investigations on the subject. Exclusion criteria were: articles in duplicate; no available summaries; not empirical; not assessing any component of body image; the sample did not consider the target age of this research (0 to 12 years old) and/or considered clinical populations; besides articles not fully available.

**Data synthesis:**

7,681 references were identified, and, after the exclusion criteria were implemented, 33 studies were analysed. Results showed that the perceptual and attitudinal dimensions focusing on body dissatisfaction were explored, mainly evaluated by silhouette scales. Intervention programs were developed internationally to prevent negative body image in children.

**Conclusions:**

The studies included in this review evaluated specific aspects of body image in children, especially body perception and body dissatisfaction. The creation of specific tools for children to evaluate body image is recommended to promote the psychosocial well being of individuals throughout human development.

## RESUMO

**Objetivo:**

Analisar a literatura científica referente à avaliação da imagem corporal em crianças por meio de uma revisão integrativa da literatura.

**Fontes de dados:**

Foi realizado um cruzamento das palavras-chave *“body image”* AND *“child”* nas bases de dados Scopus, Medline e Biblioteca Virtual de Saúde. A pesquisa eletrônica foi feita com base nos estudos publicados de janeiro de 2013 até janeiro de 2016, com o intuito de verificar os mais atuais sobre o tema. Os critérios de exclusão foram: estudos em duplicata; cujos resumos não estivessem disponíveis; não empíricos; que não avaliassem algum componente da imagem corporal; cuja amostra não considerasse a idade alvo da presente pesquisa (crianças de 0 a 12 anos); que considerassem populações clínicas; e estudos não disponíveis na íntegra.

**Síntese dos dados:**

Foram identificadas 7.681 referências e, após critérios de exclusão, 33 estudos foram analisados. Os resultados apontaram que as dimensões perceptiva e atitudinal, com foco na insatisfação corporal, têm sido exploradas, sendo avaliadas principalmente por escalas de silhuetas. Programas de intervenção foram desenvolvidos internacionalmente para prevenir o desenvolvimento de imagem corporal negativa em crianças.

**Conclusões:**

As pesquisas incluídas na presente revisão avaliaram aspectos específicos da imagem corporal de crianças, especialmente a percepção corporal e a insatisfação corporal. Recomenda-se o desenvolvimento de instrumentos específicos para crianças que busquem avaliar diferentes dimensões da imagem corporal, tendo em vista a promoção do bem-estar psicossocial dos indivíduos ao longo de todo o desenvolvimento humano.

## INTRODUCTION

Body image is understood as the “figuration of our body formed in our mind.”^1^ It is a complex and multifaceted construct, ^2^ ^,^ ^3^ which is subdivided in two dimensions: perceptual and attitudinal.^2^ ^,^ ^3^ The former defines accuracy in judgment of body size, shape, and weight.^4^ The latter involves thoughts, feelings, and behaviors related to the body.^2^ Body dissatisfaction is a component of the attitudinal dimension and refers to the negative subjective assessment of one’s body.^2^ ^,^ ^3^

The increasing number of body image studies in recent years is remarkable, especially those focusing on the adolescent and young adult population.^3^ However, little attention has been dedicated to childhood.^5^ ^,^ ^6^ According to Papalia and Feldman,^7^ childhood comprises the period from birth to the onset of puberty/adolescence. Therefore, the conclusion of this age group is not clearly defined and may vary from individual to individual. In addition, the World Health Organization^8^ uses the chronological criteria to define the stages of life, and the end of childhood is at around 10 years of age. This phase is considered of extreme relevance, since this period is configured as the basis of human formation and body image.^6^

Throughout life, body image is in permanent (de)construction.^9^ It is during childhood that weight concerns, body-related beliefs and behaviors directed at improving physical appearance may begin.^5^ ^,^ ^6^ Thus, since an early age, the individual in search of an ideal body may have his or her body image affected. It is noted that having a negative body image during childhood may be a risk factor for the development of psychopathologies in later ages.

In this sense, given that a negative body image at early ages can impact the individual’s psychological well-being in the next stages of human development, being associated with eating disorders, it becomes necessary to improve the knowledge about this subject. Thus, the objective of the present study was to analyze the scientific production regarding the evaluation of body image in children through an integrative review of the literature.

## METHOD

An integrative review of the literature was performed according to the definition of Souza, Silva and Carvalho.^10^ An electronic search of articles indexed in three databases (Scopus, Medline and Virtual Health Library - BVS) was carried out. We chose to cross the descriptors “child” AND “body image” (both indexed in the system of Descriptors in Health Science - DeCS). These descriptors are believed to reflect the objective of this review in an integral way, since the objective is to analyze the studies carried out with respect to the “body image” of “children”. It should be noted that in all databases, the terms were inserted only in English, since a greater number of findings were identified in that language during an initial search with the two sets of keywords (in Portuguese and in English). In addition, the references found by the Portuguese set were repeated in the set of English searches, since they were identified by the existence of keywords and/or abstract in English. Therefore, the authors chose to narrow the searches to English only.

As for the temporal cut, it was decided to limit the date of publication starting in January 2013. This decision was taken considering that since the 1980s, studies on body image have multiplied exponentially.^3^ ^,^ ^11^ In addition, the book Body Image: Reflections, Guidelines and Research Practices^3^, considered a landmark for studies in this field, synthesizes previous information in the literature on the subject by the year 2013. Thus, there is a broad need for a contemporary and updated approach on the theme in question. In this sense, for this review to present the most current studies on the topic, all searches were conducted in January 2016.

The process for selecting articles followed seven exclusion criteria:

1. articles in duplicate;
2. abstracts that were not available;
3. that do not use empirical methodology (review studies, critical comments etc.);
4. that did not evaluate any component of body image;
5. when the sample did not consider the age group of the present study (children from 0 to 12 years old);
6. that considered clinical populations of children (children with cancer, who have suffered burns etc.);
7. not available in full, even after searching with the help of the CAPES Newspaper Portal.

After the articles were refined from the established criteria, the manuscripts included were read in full and analyzed as to their authorship, country of research and year of publication, method employed, sample characteristics, assessed body image aspect and evaluation instrument of the body image used. Then, from the results of the studies found, we chose to group the findings into themes: “Body dissatisfaction in children” and “Body perception in children” and “Other elements related to body image”. This grouping considered the aspect of body image assessed by the studies.

## RESULTS

**Figure 1:**
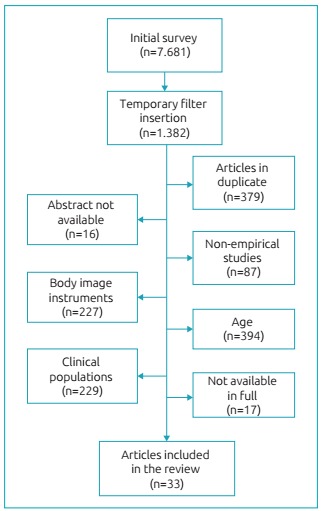
Selection of articles included in the study.

Figure 1 shows the selection of studies for analysis in the present review. It is important to mention that the initial survey identified 7,681 references. After the application of the exclusion criteria, 33 publications were analyzed.

Table 1 presents a detailed description of the main studies found in this review.^12^ ^,^ ^13^ ^,^ ^14^ ^,^ ^15^ ^,^ ^16^ ^,^ ^17^ ^,^ ^18^ ^,^ ^19^ ^,^ ^20^ ^,^ ^21^ ^,^ ^22^ ^,^ ^23^ ^,^ ^24^ ^,^ ^25^ ^,^ ^26^ ^,^ ^27^ ^,^ ^28^ ^,^ ^29^ ^,^ ^30^ ^,^ ^31^ ^,^ ^32^ ^,^ ^33^ ^,^ ^34^ ^,^ ^35^ ^,^ ^36^ ^,^ ^37^ ^,^ ^38^ ^,^ ^39^ ^,^ ^40^ ^,^ ^41^ ^,^ ^42^ ^,^ ^43^ ^,^ ^44^The following were highlighted: authors; country; year of publication; method employed; characteristics of the sample; size of the assessed body image; and evaluation instrument used. It should be noted that a sample size varied from 25 to 11,466 children.

**Table 1:**
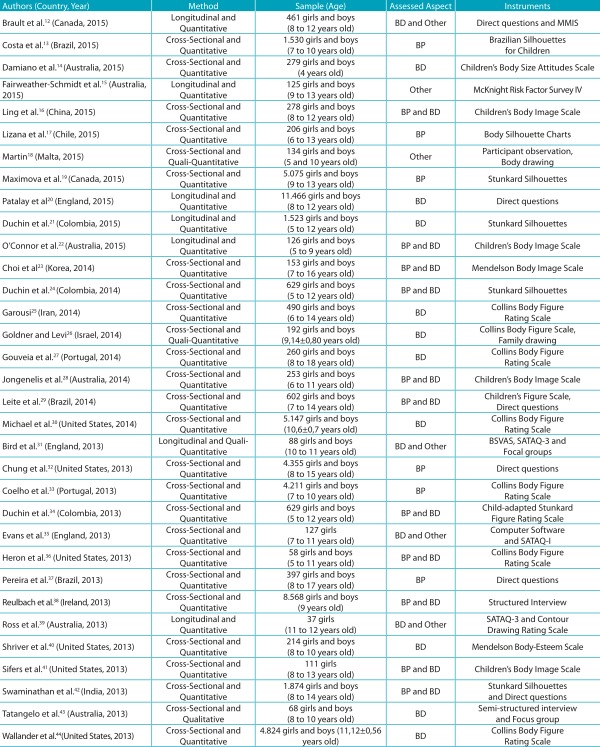
Studies on the body image of children, published from 2013 to 2016.

BP: Body perception; BD: Body dissatisfaction; SATAQ: *Sociocultural Attitudes Towards Appearance Questionnaire;* MMIS*: Multidimensional Media Influence Scale;* BSVAS*: Body Satisfaction Visual Analogue Scale*

Table 2 summarizes the information about the country of origin of the study, methods used, assessed body image aspect, applied instruments and limitations. The absolute and relative frequencies were calculated to allow the direct visualization of the most recurrent information. With respect to the country of publication, the United States and Australia are the most outstanding ones, with six studies each. Cross-sectional and quantitative methods were employed in 26 and 29 studies, respectively. The aspects of body dissatisfaction and perception were responsible for most of the studies performed, and the silhouette scales were the most frequently used instrument.

**Table 2:**
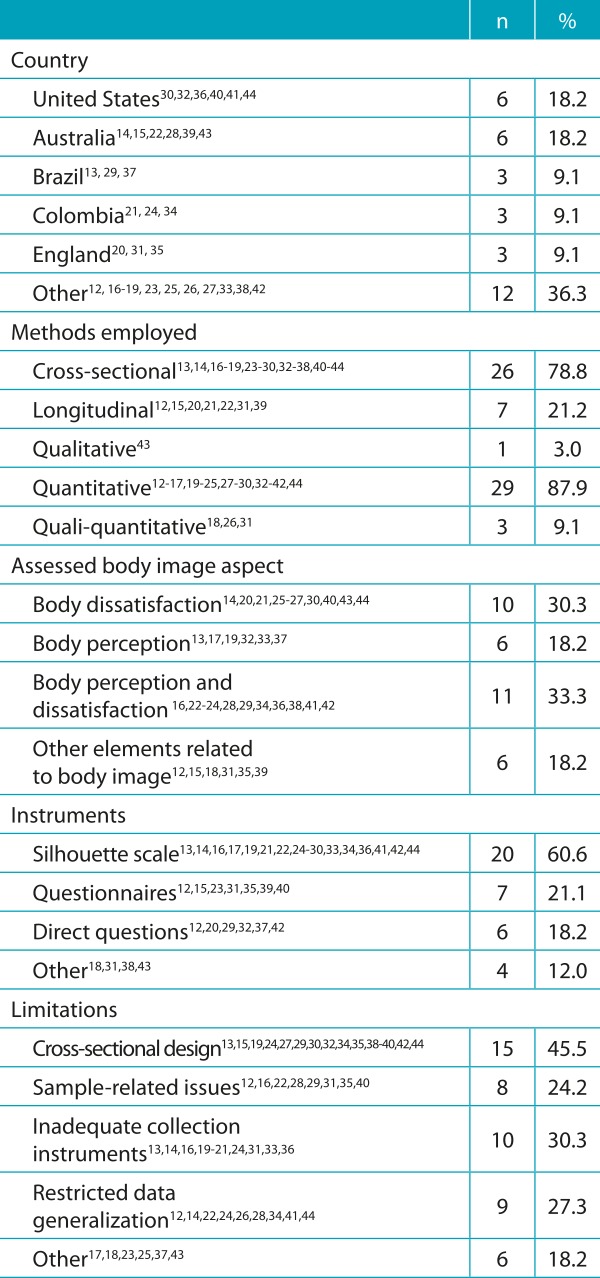
Characterization of the studies considered by absolute and relative frequency.

## DISCUSSION

The objective of the present study was to analyze the scientific production related with the evaluation of body image in children through an integrative review of the literature. This evaluation is important because, in terms of general health, if concerns about weight and body shape affects the younger ones, they could compromise psychological aspects throughout other stages of life.^23^ After analyzing the selected articles, it is necessary to mention some studies regarding sample characteristics, countries of the studies, methods, instruments used and body image size assessed in relation to contemporary scientific production on the evaluation of body image in children.

Regarding sample characteristics, it was noticed that there was great variability in the number of children included in the investigations. The design of the study may have contributed to this great disparity, since quantitative and cross-sectional analyses generally have a larger sample than qualitative and longitudinal ones. Epidemiological studies require a larger contingent sample than longitudinal or randomized controlled analyses. Still on the sample, it is observed that most evaluations assessed children in the third childhood (from 6 years of age to the beginning of adolescence).^7^ In this period, self-concept becomes more complex,^7^ and children are already able to establish comparisons between themselves and others, influencing their physical/athletic ability and physical appearance, which are the basis for the development of body image.^6^ In addition, it is during this stage that the literacy process occurs, which makes it easier to apply and understand research instruments.

Regarding the countries of the studies, it is noteworthy that Brazil has presented a restricted number of publications, less than 10%. The United States and Australia maintain a prominent position in the number of surveys conducted, accounting for 18.2% each. Therefore, national investigations on the subject are recommended. When considering the research method, it is possible to observe that the researchers of the field have prioritized quantitative (87.9%) and cross-sectional (78.8%) methods, showing the scarcity of qualitative and longitudinal investigations.

As for the instruments used, the silhouette scales were present in 60.6% of the studies performed. However, one of the main limitations pointed out by the surveys was the use of invalidated collection instruments for the populations of interest.^13^ ^,^ ^14^ ^,^ ^16^ ^,^ ^19^ ^,^ ^20^ ^,^ ^21^ ^,^ ^24^ ^,^ ^31^ ^,^ ^33^ ^,^ ^36^ Other limitations were also reported. For example, the cross-sectional design, ^13^ ^,^ ^15^ ^,^ ^19^ ^,^ ^24^ ^,^ ^27^ ^,^ ^29^ ^,^ ^30^ ^,^ ^32^ ^,^ ^34^ ^,^ ^35^ ^,^ ^38^ ^,^ ^39^ ^,^ ^40^ ^,^ ^42^ ^,^ ^44^ because it precludes causal inferences. Sample-related problems (such as small sample size, sample loss or non-representativeness of the sample) were also presented by some researchers.^12^ ^,^ ^16^ ^,^ ^22^ ^,^ ^28^ ^,^ ^29^ ^,^ ^31^ ^,^ ^35^ ^,^ ^40^ Finally, restricted data generalization has also been highlighted. ^12^ ^,^ ^14^ ^,^ ^22^ ^,^ ^24^ ^,^ ^26^ ^,^ ^28^ ^,^ ^34^ ^,^ ^41^ ^,^ ^44^ The identification of these limiting factors generates suggestions for future studies in order to fill these gaps. Thus, it is possible to propose longitudinal analyses in samples of children, using specific instruments for this population, with large and representative samples, allowing a generalization of the results.

Another factor that should be considered is the dimension of body image evaluated. From the total number of analyzed studies, 30.3% reported evaluating the affective component of the attitudinal dimension of body image, which was generally denominated “body dissatisfaction”. The perceptive dimension, which the authors referred to as “body perception”, was recurrent in 18.2% of the investigations. Several studies (33.3%) proposed to evaluate more than one dimension of body image simultaneously, especially “body perception and dissatisfaction”. “Other elements related to body image” were evaluated in 18.2% of the analyses, such as the understanding of children regarding their body shape and the internalization of the thinness ideal.

The results found in the articles were discussed afterwards, and were chosen by being divided into categories according to the body image dimensions that were evaluated by the studies. Therefore, the following topics were created: “Body dissatisfaction in children”, “Body perception in children” and “Other elements related to body image”.

### Body dissatisfaction in children

From the studies by Leite et al.^29^ and Ling et al.,^16^ which were based on silhouette scales, most of the children evaluated were dissatisfied with their body image. In contrast, when asking children directly about their satisfaction with their own body, Patalay, Sharpe, and Wolpert^20^ found a low frequency of negative body image in girls and boys. It is possible that these differences are due to the research methodology used. Gardner and Brown^45^ point out that silhouette scales tend to overestimate the values ​​of dissatisfaction found. Moreover, this instrument allows the individual to choose only one silhouette, so that he or she classifies oneself as satisfied only if it is possible to identify the same figure as ideal and real. Thus, these results indicate that the evaluation method used may influence the prevalence of body dissatisfaction and should be analyzed with caution.

Regarding the difference of dissatisfaction between sexes, Garousi^25^ and Jongenelis et al.^28^ identified that girls presented themselves as more dissatisfied than boys. Ling et al.^16^ point out that girls wanted a leaner silhouette and boys wanted a larger body. Garousi^25^ found lipophobic attitudes significantly related to the Body Mass Index (BMI) in girls. According to the literature, these results are not unique to childhood. Adolescents and female adults also tend to be dissatisfied with their body image, especially regarding body fat.^46^ ^,^ ^47^ This is especially owed to the cultural tendency to consider thinness as the ideal body pattern for females.^47^ For males, muscularity is the desired pattern.^48^ The negative evaluation of the body occurs due to the difficulty of fitting in these models.

It is already widely known in the literature that body dissatisfaction is directly related to BMI.^2^ ^,^ ^3^ The present review also confirmed this association in the infantile public: body dissatisfaction is higher among overweight individuals.^12^ Obese Australian children were more dissatisfied when compared to those with adequate weight.^28^ Leite et al.^29^ and Wallander et al.^44^ confirm these findings in Brazilian and American children, respectively. It seems that being overweight is a worry that also afflicts the younger ones.

Only one investigation included in the present review verified the relationship of body image in different ethnicities. The findings of Heron et al.^36^ did not point out racial differences in body dissatisfaction. However, Fortes et al.^5^ emphasized there is still controversy about the relationship between ethnicity and rates of body dissatisfaction in the infant population. In this sense, research is recommended to clarify the influence of racial elements on the body image of children.

Currently, the most used theoretical model for the study of body image development is that proposed by Thompson et al.,^49^ in which sociocultural factors - especially the media, parents and friends - can influence in the search for an ideal body. In the present review, we found a study in which the sociocultural model was tested in girls aged from 7 to 11 years old.^35^ According to the authors, the internalization of an ideal lean body increases the risk of eating disorders through body dissatisfaction, dietary restriction, and depression. Thus, the media was characterized as a strong influence factor in the development of deleterious health behaviors in girls. Supporting this idea, Tatangelo and Ricciardelli^43^ showed that whereas boys admired the body of male athletes, famous actresses and female singers were considered an ideal body for girls, highlighting the role of the media in body dissatisfaction.

The influence of parents was evaluated in some articles of the present review. Studies have indicated that maternal body dissatisfaction was positively related to children’s BMI gain.^21^ ^,^ ^24^ Damiano et al.^14^ showed that attitudes regarding the boys’ body size were associated with paternal body image, whereas in girls the desire for thinner figures was related to a maternal food restriction. In this sense, Michael et al.^30^ concluded that the eating habits of the mother and the father were associated respectively with the body self-esteem of girls and boys. It is also worth noting the research of Swaminatha et al.,^42^ according to which children considered by their parents as overweight or obese showed high propensity to try and lose weight. It is possible that parents have a particular importance on the body image of their children, and therefore should be taken into account in research with children - even if this influence is not perceived by them.

In addition to the media and parents, friends also have a prominent place in the sociocultural influence model.^49^ In the present review, some studies have related children’s body dissatisfaction with peer influence.^30^ ^,^ ^43^ For Michael et al.,^30^ both for boys and for girls, staying with peers and the fear of negative peer evaluation were directly related to body self-esteem. According to Tatangelo and Ricciardelli,^43^ friends helped children reinforce and criticize media messages. So, it is possible to infer that friends are fundamental in this relationship.

### Body perception in children

Some studies were carried out to verify the accuracy in the judgment of body dimensions of children. Costa et al.^13^ and Ling et al.^16^ found prevalence of high inaccuracy in Brazilian and Chinese children, respectively. According to Costa et al.,^13^ most of them overestimated their body size. Ling et al.^16^ pointed out that most of their sample underestimated their body image. The importance of accuracy in estimating body size in this age group is highlighted, as it may be the first step towards adopting healthier lifestyles.^13^

According to some investigations, various factors can influence the evaluation of body perception. Weight was determinant in the studies of Maximova et al.^19^ and Lizana et al.,^17^ which verified that boys and girls with excess weight and obesity erroneously assessed their body size, underestimating it. Other studies pointed out that children with normal weight had difficulty in perceiving their actual size or even considered themselves too fat.^37^ Thus, we understood that, as already pointed out by Duchin et al.,^24^ weight is associated with perception of body image in children.

Age was another factor that contributed with children’s body perception, as in the study by Duchin et al.,^24^ who verified that the silhouette choice was positively associated with the children’s age. Chung, Perrin, and Skinner^32^ also found that older children perceived their weight status more accurately. The authors further emphasized that girls and boys of all ages who perceived themselves to be overweight were more likely to engage in weight-loss behaviors. Thus, in children, it is appropriate to consider age when the focus is on body perception.

Differences between sexes were also identified regarding the children’s body accuracy, and the percentage of girls who were fatter was higher than that of boys.^16^ In addition, girls chose a leaner silhouette as the desired body image.^24^ It is possible that this result will interfere with weight-loss behaviors so that girls are more likely to engage in this kind of deleterious health behavior.^5^ ^,^ ^6^ It seems that girls have a harder time dealing with judging their weight and their body dimensions.

It is worth emphasizing there is still a discussion about the perceptual evaluation of body image. In the present study, we chose to present the denomination of the “assessed body image aspect” used by the authors of each article. However, misconceptions have been found regarding the appropriate concepts. That is, in some cases, the authors report evaluating “body perception”, when in fact they use instruments considered more sensitive to the evaluation of “body dissatisfaction”, such as the silhouette scales.^13^ ^,^ ^17^ Laus et al.^11^ had already indicated the presence of this conceptual confusion in the Brazilian context. This research confirmed that this also occurs in the international scenario.

According to Gardner,^50^ silhouette scales have been developed to measure body size distortion. The validity or trustworthiness of perceptual evaluation is only possible when measuring the difference between the actual size of the individual and his or her judgment of their own body size.^50^ Neves, Morgado and Tavares^51^ draw attention to the fact that the evaluation of the perceptual dimension of body image is considered to be more appropriate when technological devices of body image distortion are used for the research subjects, such as photos or filming. Thus, deeper knowledge of the body image construct to be investigated is essential in order to produce reliable results. However, based on the studies, it seems that some authors report an evaluation of this aspect of body image, even without doing it properly. More caution is required when evaluating this construct. It is suggested that future studies bring adequate devices for the real evaluation of body perception in children.

### Other elements related to body image

On a smaller scale, some investigations have evaluated other elements related to body image. Martin^18^ evaluated the understanding of children regarding their body shape through drawings and comments about their body. The author identified that obese 5-year-old girls and boys seem to be unaware of any differences in body shape. This situation changes in the group of 10-year-olds, in which excess weight is stigmatized negatively. In this study, it is pointed out that obese children develop coping strategies to deal with physical handicaps, insults and exclusion by their peers.

The internalization of the thinness ideal was further approached by Brault et al.,^12^ Bird et al.,^31^ Ross, Paxton and Rodgers^39^ and Evans et al.^35^ It seems that girls with normal BMI or overweight tend to report more pressure to be thin in comparison to underweight girls.^12^ Low-weight boys reported more awareness of the norms of the thinner ideal than other boys.^12^ Possibly the lean-body ideal propagated by the media affects boys and girls of school age.

## FINAL CONSIDERATIONS

The studies included in this review pointed out the need for intervention programs in order to prevent the development of negative body image in children.^12^ ^,^ ^43^ Some analyses have already been developed with this purpose and indicated positive results.^15^ ^,^ ^22^ ^,^ ^31^ ^,^ ^39^ ^,^ ^41^ Some of the main findings of these studies are: improvement in body satisfaction; ^22^ ^,^ ^31^ decreasing concern about weight and body shape;^15^ decrease in discrepancy between real and ideal body image;^41^ reduction of the internalization of cultural-looking ideals;^31^ ^,^ ^39^ reduced body comparisons and improved self-esteem.^39^ From this perspective, the benefits of intervention programs are recognized and initiatives such as these should be encouraged.

From the studies included in the present review, we conclude that research has been done to evaluate the body image of children, especially regarding the perceptual and attitudinal dimensions, focusing on body dissatisfaction. In a smaller scale, investigations were found aiming at evaluating other elements related with body image. Perhaps this is due to the scarcity of instruments on these components for children. Thus, studies are recommended in order to create or validate scales for children, looking for a global understanding of infantile body image. Finally, it is worth emphasizing that studies that evaluate the body image in children will bring benefits to the mental health of individuals throughout human development.
